# Comparison of carotid plaque tissue characteristics in patients with acute coronary syndrome or stable angina pectoris: assessment by iPlaque, transcutaneous carotid ultrasonography with integrated backscatter analysis

**DOI:** 10.1186/s12947-015-0031-6

**Published:** 2015-07-25

**Authors:** Mika Bando, Hirotsugu Yamada, Kenya Kusunose, Daiju Fukuda, Rie Amano, Rina Tamai, Yuta Torii, Yukina Hirata, Susumu Nishio, Koji Yamaguchi, Takeshi Soeki, Tetsuzo Wakatsuki, Masataka Sata

**Affiliations:** Department of Cardiovascular Medicine, Tokushima University Hospital, 2-50-1 Kuramoto, Tokushima, Japan; Ultrasound Examination Center, Tokushima University Hospital, Tokushima, Japan

**Keywords:** Carotid arteries, Plaque vulnerability, Ultrasound, Acute coronary syndrome (ACS)

## Abstract

**Background:**

The association of the tissue characteristics of carotid plaques with coronary artery disease has attracted interest. The present study compared the tissue characteristics of carotid plaques in patients with acute coronary syndrome (ACS) with those in patients with stable angina pectoris (SAP) using the iPlaque system, which is based on ultrasound integrated backscatter.

**Methods and results:**

Carotid ultrasound examinations were performed in 26 patients with ACS, and 38 age- and gender-matched patients with SAP. Neither plaque area nor maximal intima-media thickness differed significantly between the two groups. However, the average integrated backscatter value within the plaque was greater in the ACS patients than in the SAP patients. iPlaque analysis revealed that the percentage blue area (lipid pool) was greater in the ACS patients than in the SAP patients (43.4 ± 11.2 vs 18.3 ± 10.3 %, p < 0.0001), and that the percentage green area (fibrosis) was lower in the ACS than in the SAP patients (7.5 ± 7.5 % vs 20.7 ± 11.7 %, p < 0.0001).

**Conclusions:**

The lipid component of carotid plaques is greater in ACS patients than in SAP patients. Our iPlaque system provides a useful and feasible method for the tissue characterization of carotid plaques in the clinical setting.

## Background

Recent developments in imaging technology, such as intravascular ultrasound (IVUS) and optical coherence tomography, enable us to obtain information on coronary plaques in patients [1, 2].Through numerous investigations using these imaging devices, it has become recognized that a lipid-rich plaque in the coronary artery is vulnerable, and can precede the development of acute coronary syndrome (ACS) [3–6]. Quantitative analysis of tissue characteristics of coronary plaques has been achieved by IVUS with integrated backscatter (IB) ultrasound analysis [7] and this technique has been used in large multicenter clinical studies [8]. IB-IVUS enables the identification of the histological features of coronary plaques, such as lipid pools, fibrous tissue, and calcified tissue [9].

The tissue composition of carotid plaques has been reported to predict the risk of cerebrovascular and cardiovascular events [10–12]. Patients with ACS were found to have unstable atherosclerotic plaques in carotid arteries [13, 14]. In carotid ultrasonography, the video density of plaque measured as gray scale median has been used to quantify the tissue characteristics of plaques. [15–17] Some investigators have also applied the IB ultrasound technique to carotid plaques [18–20] however, it has not been used clinically.

To transfer this knowledge to the clinical setting, we have developed the iPlaque system [21], an ultrasound IB-based image analysis software that enables the tissue components of carotid plaques to be visualized with high resolution in a color-coded display. It also quantifies the proportions of each tissue component in the plaque. The system provides tissue characterization similar to that attainable with IB-IVUS, which is based on percutaneous carotid ultrasonography. It has been validated by comparison with the results of histology of samples of plaques taken from patients during carotid endarterectomy (CEA) [22].

The purpose of this study was to compare the tissue characteristics of carotid plaques in patients with ACS with those in patients with stable angina pectoris (SAP) using our iPlaque system, which eventually establishes one of the clinical applications for the system.

## Methods

### Subjects

From 195 patients who had undergone percutaneous coronary intervention and carotid ultrasonography in our department from January 2010 to June 2014, patients with suitable carotid plaques for iPlaque analysis were enrolled in this study. The selection criteria for suitable iPlaque analysis were as follows: (1) maximal intima-media thickness (IMT) of the plaque ≥ 1.5 mm, (2) located in either the common or internal carotid artery, (3) located in the far wall, and (4) without severe stenosis (defined as > 75 % area stenosis). Of them, 26 patients were diagnosed as having ACS, and 169 were diagnosed with SAP. The ACS group included 8 patients with ST elevation myocardial infarction, 12 patients with non-ST elevation myocardial infarction, and 4 patients with unstable angina pectoris. To compare the tissue characteristics of the carotid artery with those in the 26 ACS patients, 38 age- and gender-matched subjects were randomly selected from the SAP patients. If the true difference of average IB values of carotid plaque in the ACS and SAP is 2.0 with standard deviation 2.5 according to a previous study [13], our sample size were enough to reject the null hypothesis that the population means of the ACS and SAP groups are equal with probability (power) 0.85. The Type I error probability associated with this test of this null hypothesis is 0.05.

### Definition of diseases

ACS included acute myocardial infarction and unstable angina pectoris. The diagnosis of acute myocardial infarction was made if the patient had typical chest pain with characteristic electrocardiogram changes and elevation of at least one positive biomarker (creatinine kinase [CK], CK-MB, or troponin T). A diagnosis of unstable angina pectoris was made when there was angina with a progressive crescendo pattern, or angina that occurred at rest. SAP was defined as no episodes of angina at rest in a patient who underwent percutaneous coronary intervention more than six months previously. Hypertension was defined as a systolic blood pressure >140 mmHg and/or a diastolic pressure >90 mmHg, or current treatment with antihypertensive medication. Diabetes was defined as a fasting blood glucose ≥126 mg/dl, hemoglobin A1c ≥6.5 %, and/or the need for oral hypoglycemic agents or insulin. Hyperlipidemia was defined as plasma total cholesterol >220 mg/dl or the use of lipid-lowering therapy. Smoking was defined as current or previous smoking [23].

### Study protocols

Carotid ultrasound examinations were performed using the Logiq 7 with a 10 MHz linear-array transducer (GE Healthcare, Milwaukee, WI, USA). All carotid scans were performed by a single well-trained operator, who had no information on the clinical characteristics of the patients. The position of the probe was adjusted so that the ultrasonic beam was vertical to the artery wall, and maximal IMT was measured manually in the common carotid artery, bulb and internal carotid artery according to the standard method recommended by Japanese Society of Ultrasound in Medicine [15]. Images of longitudinal sections of carotid plaque of maximal IMT were digitally stored with RAW data. The data were analyzed off-line by our dedicated software (iPlaque), as described later. The study was in accordance with the Declaration of Helsinki, and was approved by the Institutional Review Board of the University of Tokushima. Each subject gave informed written consent.

### iPlaque analysis

In the iPlaque analysis, ultrasound attenuation was adjusted by 2.0 dB/mm at first. Then, each IB value was normalized to the calibrated value by setting the IB value of blood near the plaque to −70 dB. The edge of the plaque of interest was manually traced, and then the program calculated the plaque area and the average IB value in the plaque. The area of each tissue component was categorized in accordance with published IB thresholds, and the amount of each component as a percentage of the whole plaque area was calculated. The IB thresholds used were: −30.14 dB≦IB = calcification (display in red); −46.18 dB≦IB < −30.14 dB = dense fibrosis (display in yellow); −61.23 dB≦IB < −46.18 dB = fibrosis (display in green); − IB < −61.23 dB = lipid pool (display in blue) [22].

### Reproducibility

To assess measurement reproducibility, 20 patients with ACS were selected randomly and we evaluated two different measurements on the 20 ACS patients. The correlation between the measured values of percentage blue area by the two observers (Observer I and Observer II) was regarded as the inter-observer correlation. The correlation between the first and second values determined by one observer (Observer I) was regarded as the intra-observer correlation. The coefficients of variation of interobserver and intraobserver was 7.3 % and 6.6 %, respectively. Also, we plotted the mean differences against the mean of 2 measurements according to the methods described by Bland - Altman.

### Statistical analysis

Data are presented as mean ± SD. Data were tested for normality using the Kolmogorov-Smirnov test. Continuous variables were compared using unpaired Student’s t tests or Mann–Whitney test as appropriate, whereas categorical variables were compared using Chi-square test or Fisher’s exact test, as appropriate.　Receiver-operating characteristic (ROC) curves were generated to determine optimal cutoff values of continuous variables. Correlation coefficients were calculated by using the least-squares fit (forced through zero), and Bland - Altman analysis was used. Linear regression model assumptions of linearity, independence of observations, normality of error terms, homoscedasticity were all checked. All statistical analyses were carried out with Medcalc Software 12.7.5.0 (Ghent, Belgium). A p-value <0.05 was considered significant.

## Results

### Patients’ characteristics and conventional ultrasound parameters

**Fig. 1:**
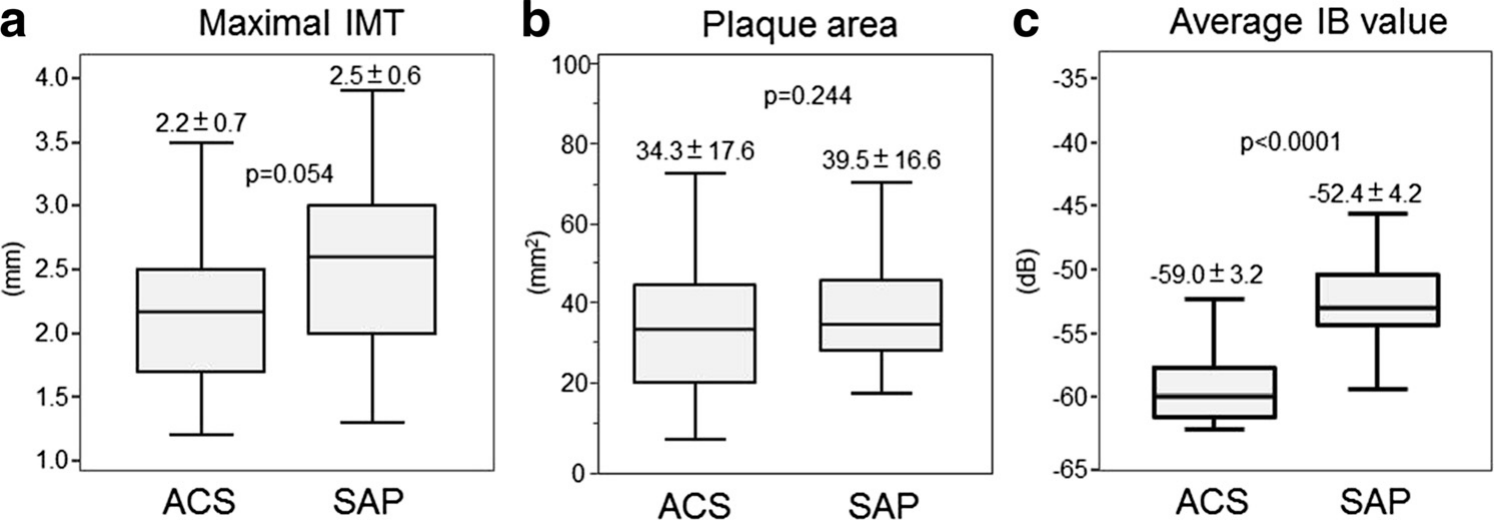
Comparisons of carotid ultrasonographic parameters (**a**:maximal intima-medina thickness, **b**:plaque area, and **c**:average integrated backscatter value) between the patients

The clinical characteristics of the two groups of patients are shown in Table 1. There were no significant differences in age, gender, body mass index (BMI), smoking, left ventricular ejection fraction (LVEF), or the prevalence of other diseases (hypertension, hypercholesterolemia, diabetes mellitus). Serum low density lipoprotein cholesterol (LDL-C) and oxidized LDL concentrations were significantly greater in patients with ACS than in those with SAP. There were no differences in medication between the two groups (including antihypertensive agents, diabetes drugs, and statins). Neither plaque area nor maximal IMT showed significant differences between the two groups (Fig. 1a, b). The average IB value within the plaque was greater in the ACS patients than in the SAP patients (Fig. 1c).

**Table 1:** Comparisons of patients’ characteristics between groups with acute coronary syndrome and stable angina pectoris

	Acute Coronary Syndrome	Stable Angina Pectoris	*p* value
	(*n* = 26)	(*n* = 38)	
Age (yrs)	67 ± 12	69 ± 7	0.63
Men (%)	18 (69)	28 (74)	0.58
Hypertension (%)	17 (65)	31 (82)	0.18
Hypercholesterolemia (%)	22 (85)	30 (79)	0.53
Diabetes mellitus (%)	12 (46)	19 (50)	0.69
Smoking (%)	20 (77)	29 (79)	0.89
BMI (kg/m^2^)	24.0 ± 3.8	24.5 ± 3.3	0.64
LVEF (%)	58 ± 10	63 ± 11	0.10
LDL-C (mg/dl)	113 ± 36	94 ± 26	0.03
HDL-C (mg/dl)	48 ± 15	54 ± 14	0.13
TG (mg/dl)	159 ± 107	121 ± 87	0.15
oxidized LDL (mg/dl)	139 ± 50	106 ± 28	0.005
eGFR (ml/min)	73 ± 22	746 ± 18	0.672
hs CRP (mg/l)	0.92 ± 1.89	0.14 ± 0.21	0.061
TG (mg/dl)	159 ± 107	121 ± 87	0.145

*BMI* body mass index, *LVEF* left ventricular ejection fraction, *LDL-C* low-densityllipoprotein cholesterol, *HDL-C* high-density lipoprotein cholesterol, *TG* triglyceride, *hs CRP* high sensitive C reactive protein

### Percentage tissue composition in carotid plaque

**Fig. 2:**
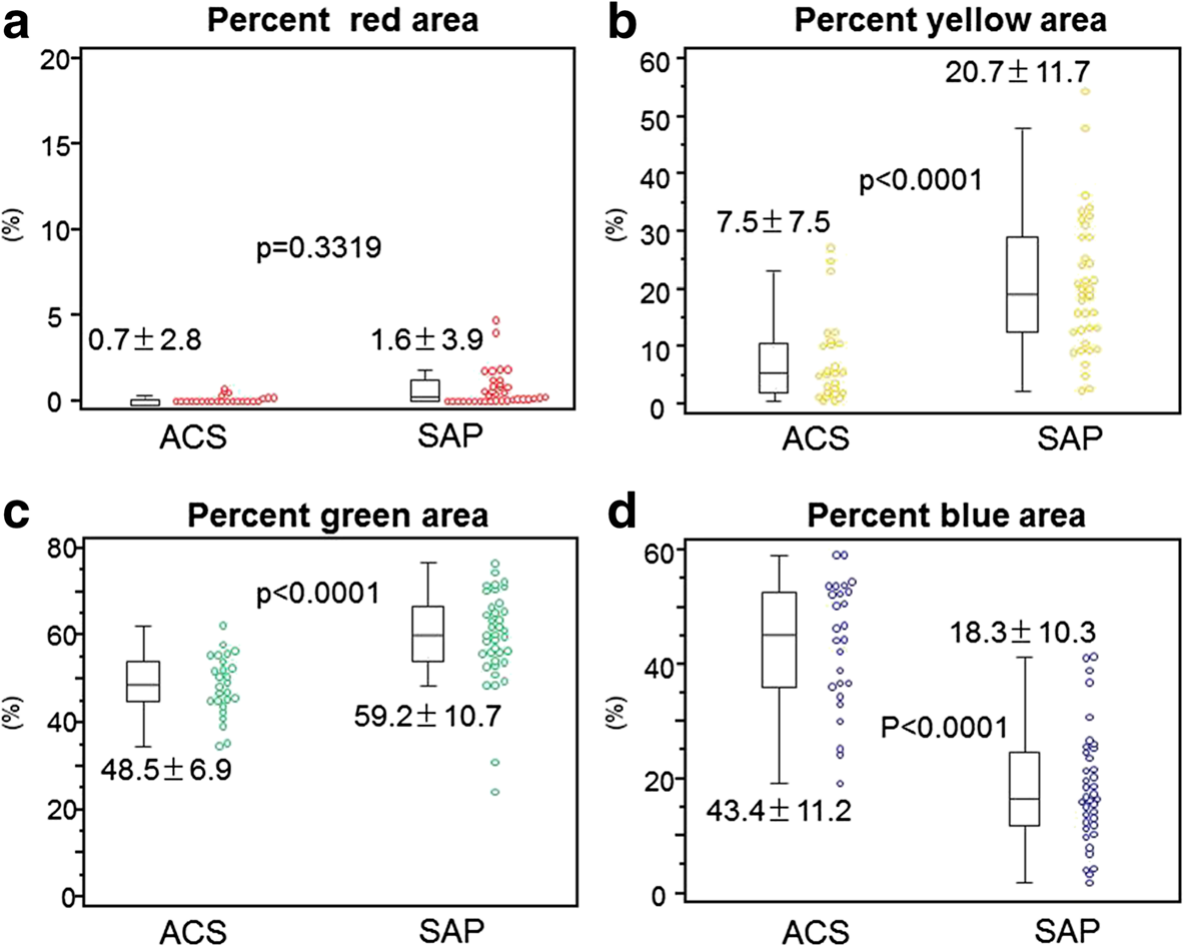
Comparisons of each tissue component (**a**:percent red area, **b**:percent yellow area, **c**:percent green area, and **d**:percent blue area) of carotid plaque determined by the iPlaque analysis in patients with acute coronarysyndrome (ACS) and stable angina pectoris (SAP): Mean ± SD is displayed

**Fig. 3:**
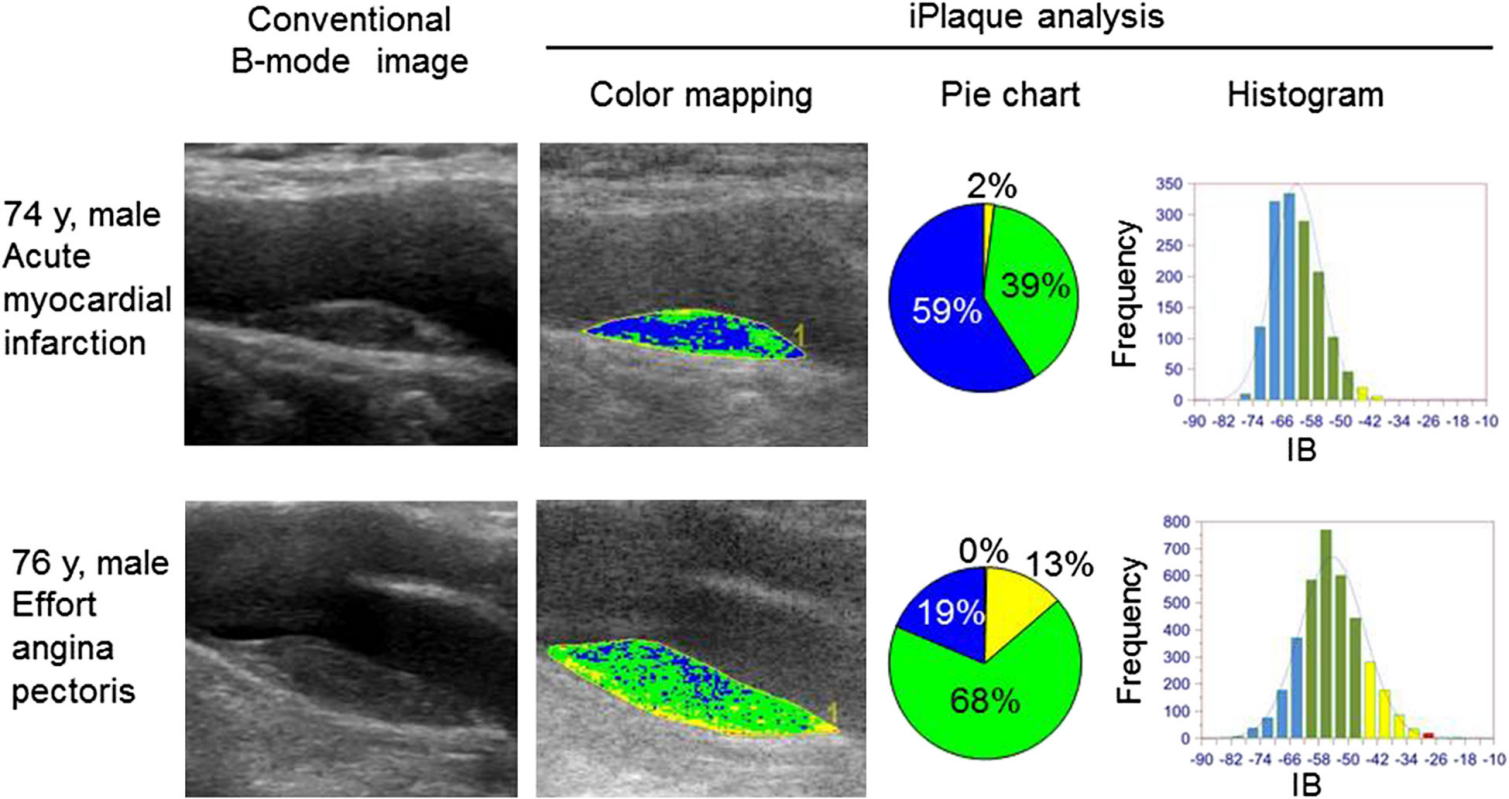
Conventional B-mode images and iPlaque analysis of carotid plaque in representative patients with acute coronary syndrome and stable angina pectoris

The iPlaque analysis revealed that the blue area (expressed as a percentage) was significantly greater in the ACS patients than in the SAP patients (Fig. 2d). The yellow and green areas were significantly smaller in the ACS than in the SAP group, and the red area was not significantly different between the two groups (Fig. 2a, b, c). Conventional B-mode image and color mapping of tissue characterization by iPlaque of carotid plaques in representative cases in both groups are shown in Fig. 3. In these cases, the blue area was 59 % in the ACS patient and 20 % in the SAP patient.

### Parameters of iPlaque analysis for identifying ACS

**Fig. 4:**
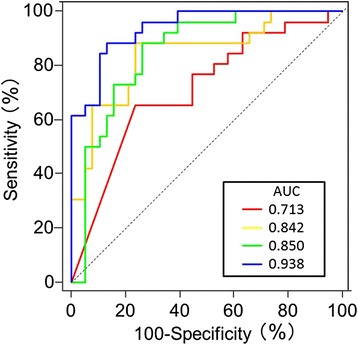
Receiver operating characteristic curves of the iPlaque analysis: Each color of the graph corresponds to a different tissue component in the carotid plaque, as described in the text

Receiver operating characteristic (ROC) curves for identifying ACS in all subjects using iPlaque parameters were constructed (Fig. 4). A blue area > 26.6 % was determined as the best criterion for identifying ACS with 88 % sensitivity and 87 % specificity. The area under the curve for each parameter was 0.938 for percentage blue, 0.850 for green, 0.842 for yellow, and 0.713 for red. For diagnosing ACS, the percentage blue area was better than the percentage red and yellow (*p* < 0.0001, *p* = 0.0180, respectively), and also tended to be better than the percentage green (*p* = 0.0606).

### Reproducibility

**Fig. 5:**
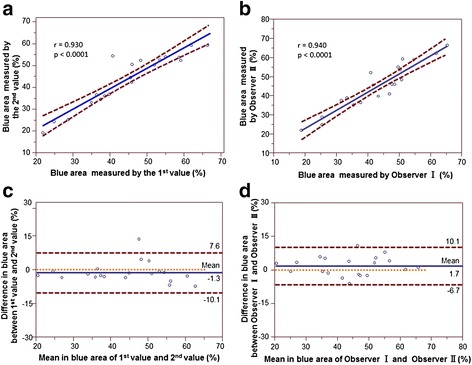
Linear regression analysis and Bland-Altman analysis of blue area measured by one of the observers and two observers. **a**:correlation between the first and the second measurement of blue area. **b**:correlation between the blue area measured by the first observer and the second observer. **c**:association between the mean value and the difference in blue area measured twice by one observer. **d**:association between the mean value and the difference in blue area measured by the first and the second observers

The correlation between the first and second measurements by one of the observers was good (r = 0.930, *p* < 0.0001). The correlation between the measured values of percent blue area by two observers (r = 0.940, *p* < 0.0001) was also good, which was similar with previous reports [24, 25]. Bland Altman plots for reproducibility of blue area by two observers showing a mean difference (solid line) of −1.3 %, and Bland Altman limits (dashed lines) of +7.6 and −10.1. The solid red line denotes bias (mean of differences) and the dashed lines denote 95 % limits of agreement (2 SD of the difference). Also, Bland Altman plots for reproducibility of blue area measured by one of the observers showing a mean difference (solid line) of +1.7, and Bland Altman limits (dashed lines) of +10.1 and −6.7 (Fig. 5).

## Discussion

In the present study, we have examined the tissue characteristics of carotid plaques in patients with ACS or SAP, using the recently developed iPlaque imaging analytical system based on ultrasound IB data. The iPlaque images had enough resolution quality to analyze the tissue characteristics. We found that the percentage blue area of plaque was greater in ACS patients than in SAP patients. Thus, we have confirmed that patients with ACS have vulnerable plaques, and that SAP patients have plaques that are more fibrous and therefore more stable. The study also established the usefulness and feasibility of the iPlaque system for quantifying carotid plaque tissue characteristics in the clinical setting.

### Relationship between carotid atherosclerosis and cardiac events

A strong association between coronary artery disease and carotid atherosclerosis has been reported by many investigators. As both arteries share the same systemic environment, atherosclerotic changes in the two sites are believed to occur to a similar degree. It has been reported that IMT was significantly associated with the presence of coronary artery disease, and the measurement has been shown to be an independent predictor of future coronary events [26]. Furthermore, the echogenicity of carotid artery plaque has been reported to be a predictor of the future risk of developing ACS [15, 18, 19]. Seo et al. reported that echolucent carotid plaques evaluated by the gray scale median were strongly associated with ACS [18]. Two studies have evaluated the IB values of carotid plaques and assessed its relationship with coronary complications in patients with coronary artery disease [19, 27]. Both found that low IB carotid plaque was associated with an increased risk of future events. Also, several studies indicate the usefulness of carotid IB values to detect the unstable plaques and the effect of statins [28, 29]. The iPlaque images were shown as color-coded and we could use this system quantitative assessment of plaque vulnerability.

### Ultrasound tissue characterization imaging of carotid plaque

Evaluation of tissue characteristics of carotid plaque has been endeavored by IB since 1983 [30]. or using gray-scale median since 1989 [31]. In these pioneer studies, investigators analyzed average gray-scale median or IB value of the one ultrasonographic section of carotid plaque [27, 28]. Consequently, color code mapping of tissue contents in carotid plaque based of gray-scale median [16, 32] and IB value [9] has been attempted. Because the basic quality of ultrasound B-mode image has been significantly in these days by technological innovation, our iPlaque analysis can provide much better spatial resolution compared to previous studies. Our system also achieved automatic imaging processing using custom-made software, while previous method needed manual calculation of IB value in each region of interests in the plaque.

### Clinical implications

As the tissue characterization of coronary artery plaque needs IB-IVUS, which requires catheterization, tissue characterization of carotid plaque may be used as a non-invasive surrogate procedure. Our results suggest that patients with vulnerable coronary plaques may also have vulnerable plaques in their carotid arteries. As iPlaque analysis of carotid plaque is both non-invasive and quantitative, and enables repeated measurements to be made. In addition, iPlaque analysis permits to judge the therapeutic effect of plaque stabilization easily, while IB-IVUS is an invasive technique. We may be able to prevent the occurrence of ACS in patients with vulnerable plaques using iPlaque analysis if the medication stabilizes the carotid plaques. Another application might for the prediction of the future risk of ACS in patients with life-style disease and metabolic syndrome, which is currently very difficult.

### Limitations

Our sample size was small, but it was enough for our statistical analysis. In this study, we did not examine the feasibility of our system. In consecutive patients with 36 ACS, our iPlaque analysis could be applied in 26 (72 %) patients. Feasibility of this method need to be examined in future research. We analyzed data from only one section of plaque in highly selected patients. To assess the plaque size and its tissue components more accurately, three-dimensional ultrasound should be used with multiple sections of two-dimensional images, or alternatively it would be necessary to develop a three-dimensional linear probe for carotid ultrasound examination. One of the major limitations is that a calcified lesion in the near wall of the carotid artery creates an acoustic shadow, making the analysis impossible. However, such a calcified lesion was usually observed only in a more advanced stage of atherosclerosis. Our main target population was patients in an early stage of atherosclerosis with some risk factors.

## Conclusions

Carotid plaques in ACS patients have a greater lipid component and a smaller fibrous component than plaques in SAP patients. Our iPlaque system, a non-invasive imaging algorithm based on ultrasound IB, is useful and feasible for the tissue characterization of carotid plaques in the clinical setting.

## Abbreviations

ACS: Acute coronary syndrome
SAP: Stable angina pectoris
IVUS: Intravascular ultrasound
IB: Integrated backscatter
CEA: Carotid endarterectomy
IMT: Intima-media thickness
CK: Creatinine kinase
ROC: Receiver-operating characteristic
BMI: Body mass index
LDL: Low density lipoprotein cholesterol
LVEF: Left ventricular ejection fraction
HDL-C: High-density lipoprotein cholesterol
TG: Triglyceride
hs CRP: high sensitive C reactive protein

## References

[1] Hodgson JM, Reddy KG, Suneja R, Nair RN, Lesnefsky EJ, Sheehan HM (1993). Intracoronary ultrasound imaging: correlation of plaque morphology with angiography, clinical syndrome and procedural results in patients undergoing coronary angioplasty. J Am Coll Cardiol.

[2] Kume T, Akasaka T, Kawamoto T, Ogasawara Y, Watanabe N, Toyota E, Neishi Y, Sukmawan R, Sadahira Y, Yoshida K (2006). Assessment of coronary arterial thrombus by optical coherence tomography. Am J Cardiol.

[3] Fuster V, Badimon L, Badimon JJ, Chesebro JH (1992). The pathogenesis of coronary artery disease and the acute coronary syndromes (1). N Engl J Med.

[4] Stary HC, Chandler AB, Dinsmore RE, Fuster V, Glagov S, Insull W, Rosenfeld ME, Schwartz CJ, Wagner WD, Wissler RW (1995). A definition of advanced types of atherosclerotic lesions and a histological classification of atherosclerosis. A report from the Committee on Vascular Lesions of the Council on Arteriosclerosis, American Heart Association. Circulation.

[5] Thieme T, Wernecke KD, Meyer R, Brandenstein E, Habedank D, Hinz A, Felix SB, Baumann G, Kleber FX (1996). Angioscopic evaluation of atherosclerotic plaques: validation by histomorphologic analysis and association with stable and unstable coronary syndromes. J Am Coll Cardiol.

[6] Virmani R, Kolodgie FD, Burke AP, Farb A, Schwartz SM (2000). Lessons from sudden coronary death: a comprehensive morphological classification scheme for atherosclerotic lesions. Arterioscler Thromb Vasc Biol.

[7] Sano K, Kawasaki M, Okubo M, Yokoyama H, Ito Y, Murata I, Kawai T, Tsuchiya K, Nishigaki K, Takemura G (2005). In vivo quantitative tissue characterization of angiographically normal coronary lesions and the relation with risk factors: a study using integrated backscatter intravascular ultrasound. Circ J.

[8] Hiro T, Kimura T, Morimoto T, Miyauchi K, Nakagawa Y, Yamagishi M, Ozaki Y, Kimura K, Saito S, Yamaguchi T (2009). Effect of intensive statin therapy on regression of coronary atherosclerosis in patients with acute coronary syndrome: a multicenter randomized trial evaluated by volumetric intravascular ultrasound using pitavastatin versus atorvastatin (JAPAN-ACS study). J Am Coll Cardiol.

[9] Kawasaki M, Takatsu H, Noda T, Ito Y, Kunishima A, Arai M, Nishigaki K, Takemura G, Morita N, Minatoguchi S (2001). Noninvasive quantitative tissue characterization and two-dimensional color-coded map of human atherosclerotic lesions using ultrasound integrated backscatter: comparison between histology and integrated backscatter images. J Am Coll Cardiol.

[10] Gronholdt ML, Nordestgaard BG, Schroeder TV, Vorstrup S, Sillesen H (2001). Ultrasonic echolucent carotid plaques predict future strokes. Circulation.

[11] Liapis CD, Kakisis JD, Dimitroulis DA, Kostakis AG (2002). The impact of the carotid plaque type on restenosis and future cardiovascular events: a 12-year prospective study. Eur J Vasc Endvasc Surg.

[12] Rossi M, Cupisti A, Perrone L, Santoro G (2002). Carotid ultrasound backscatter analysis in hypertensive and in healthy subjects. Ultrasound Med Biol.

[13] Rossi A, Franceschini L, Fusaro M, Cicoira M, Eleas AA, Golia G, Bonapace S, Santini F, Sangiorgi G, Zardini P (2006). Carotid atherosclerotic plaque instability in patients with acute myocardial infarction. Int J Cardiol.

[14] Lombardo A, Biasucci LM, Lanza GA, Coli S, Silvestri P, Cianflone D, Liuzzo G, Burzotta F, Crea F, Maseri A (2004). Inflammation as a possible link between coronary and carotid plaque instability. Circulation.

[15] Committee TaDC (2009). Standard method for ultrasound evaluation of carotid artery lesions. J Med Ultrasonics.

[16] Lal BK, Hobson RW, Pappas PJ, Kubicka R, Hameed M, Chakhtoura EY, Jamil Z, Padberg FT, Haser PB, Duran WN (2002). Pixel distribution analysis of B-mode ultrasound scan images predicts histologic features of atherosclerotic carotid plaques. J Vasc Surg.

[17] Baroncini LA, Pazin Filho A, Murta Junior LO, Martins AR, Ramos SG, Cherri J, Piccinato CE (2006). Ultrasonic tissue characterization of vulnerable carotid plaque: correlation between videodensitometric method and histological examination. Cardiovasc Ultrasound.

[18] Seo Y, Watanabe S, Ishizu T, Moriyama N, Takeyasu N, Maeda H, Ishimitsu T, Aonuma K, Yamaguchi I (2006). Echolucent carotid plaques as a feature in patients with acute coronary syndrome. Circ J.

[19] Honda O, Sugiyama S, Kugiyama K, Fukushima H, Nakamura S, Koide S, Kojima S, Hirai N, Kawano H, Soejima H (2004). Echolucent carotid plaques predict future coronary events in patients with coronary artery disease. J Am Coll Cardiol.

[20] Nagano K, Yamagami H, Tsukamoto Y, Nagatsuka K, Yasaka M, Nagata I, Hori M, Kitagawa K, Naritomi H (2008). Quantitative evaluation of carotid plaque echogenicity by integrated backscatter analysis: correlation with symptomatic history and histologic findings. Cerebrovasc Dis.

[21] Uematsu M, Nakamura T, Sugamata W, Kitta Y, Fujioka D, Saito Y, Kawabata K, Obata JE, Watanabe Y, Watanabe K (2013). Echolucency of carotid plaque is useful for assessment of residual cardiovascular risk in patients with chronic coronary artery disease who achieve LDL-C goals on statin therapy. Circ J.

[22] Bando M, Yamada H, Kusunose K, Fukuda D, Amano R, Tamai R et al.: Noninvasive Quantitative Tissue Characterization of Carotid Plaque Using Color-coded Mapping Based on Ultrasound Integrated Backscatter. J Am Coll Cardiol Img 2015, In press. 10.1016/j.jcmg.2015.02.017 26093930

[23] Anderson JL, Adams CD, Antman EM, Bridges CR, Califf RM, Casey DE, Chavey WE, Fesmire FM, Hochman JS, Levin TN (2007). ACC/AHA 2007 guidelines for the management of patients with unstable angina/non ST-elevation myocardial infarction: a report of the American College of Cardiology/American Heart Association Task Force on Practice Guidelines: developed in collaboration with the American College of Emergency Physicians, the Society for Cardiovascular Angiography and Interventions, and the Society of Thoracic Surgeons: endorsed by the American Association of Cardiovascular and Pulmonary Rehabilitation and the Society for Academic Emergency Medicine. Circulation.

[24] Weber A, Jones EF, Zavala JA, Ponnuthurai FA, Donnan GA (2008). Intraobserver and interobserver variability of transesophageal echocardiography in aortic arch atheroma measurement. J Am Soc Echocardiogr.

[25] Masuda J, Terashima M, Yokoyama M (2001). Improved reproducibility of intravascular ultrasound assessment of coronary in-stent neointima with the use of an echogenic contrast agent. Jpn Circ J.

[26] Bots ML, Hoes AW, Koudstaal PJ, Hofman A, Grobbee DE (1997). Common carotid intima-media thickness and risk of stroke and myocardial infarction: the Rotterdam Study. Circulation.

[27] Takiuchi S, Rakugi H, Honda K, Masuyama T, Hirata N, Ito H, Sugimoto K, Yanagitani Y, Moriguchi K, Okamura A (2000). Quantitative ultrasonic tissue characterization can identify high-risk atherosclerotic alteration in human carotid arteries. Circulation.

[28] Waki H, Masuyama T, Mori H, Maeda T, Kitade K, Moriyasu K, Tsujimoto M, Fujimoto K, Koshimae N, Matsuura N (2003). Ultrasonic tissue characterization of the atherosclerotic carotid artery: histological correlates or carotid integrated backscatter. Circ J.

[29] Ito Y, Kawasaki M, Yokoyama H, Okubo M, Sano K, Arai M, Nishigaki K, Uno Y, Takemura G, Minatoguchi S (2004). Different effects of pravastatin and cerivastatin on the media of the carotid arteries as assessed by integrated backscatter ultrasound. Circ J.

[30] Picano E, Landini L, Distante A, Sarnelli R, Benassi A, L’Abbate A (1983). Different degrees of atherosclerosis detected by backscattered ultrasound: an in vitro study on fixed human aortic walls. J Clin Ultrasound.

[31] Spagnoli LG, Mauriello A, Bonanno E, Santeusanio G, Fieschi C, Fiorani P, Zanette E (1989). Echodensitometry: a methodologic approach to the non-invasive diagnosis of carotid atherosclerotic plaques. Int Angiol.

[32] Ibrahimi P, Jashari F, Johansson E, Gronlund C, Bajraktari G, Wester P, Henein MY (2014). Vulnerable plaques in the contralateral carotid arteries in symptomatic patients: a detailed ultrasound analysis. Atherosclerosis.

